# Development and validation of a novel stem cell subtype for bladder cancer based on stem genomic profiling

**DOI:** 10.1186/s13287-020-01973-4

**Published:** 2020-10-28

**Authors:** Chaozhi Tang, Jiakang Ma, Xiuli Liu, Zhengchun Liu

**Affiliations:** 1 Department of Urology, The First Affiliated Hospital of China Medical University, Shenyang, 110001 China; 2 Department of Oncology, The Second Affiliated Hospital of Zhengzhou University, Zhengzhou, 450014 China; 3 Department of Oncology, Affiliated Hospital of Guilin Medical University, Guilin, 541001 China; 4 Department of Radiation Oncology, Affiliated Hospital of Guilin Medical University, Guilin, 541001 China

**Keywords:** Bladder cancer, Stem cells, Immune microenvironment, Hypoxia, Epithelial-mesenchymal transition, Immunotherapy

## Abstract

**Background:**

Bladder cancer (BLCA) is the fifth most common type of cancer worldwide, with high recurrence and progression rates. Although considerable progress has been made in the treatment of BLCA through accurate typing of molecular characteristics, little is known regarding the various genetic and epigenetic changes that have evolved in stem and progenitor cells. To address this issue, we have developed a novel stem cell typing method.

**Methods:**

Based on six published genomic datasets, we used 26 stem cell gene sets to classify each dataset. Unsupervised and supervised machine learning methods were used to perform the classification.

**Results:**

We classified BLCA into three subtypes—high stem cell enrichment (SCE_H), medium stem cell enrichment (SCE_M), and low stem cell enrichment (SCE_L)—based on multiple cross-platform datasets. The stability and reliability of the classification were verified. Compared with the other subtypes, SCE_H had the highest degree of cancer stem cell concentration, highest level of immune cell infiltration, and highest sensitivity not only to predicted anti-PD-1 immunosuppressive therapy but also to conventional chemotherapeutic agents such as cisplatin, sunitinib, and vinblastine; however, this group had the worst prognosis. Comparison of gene set enrichment analysis results for pathway enrichment of various subtypes reveals that the SCE_H subtype activates the important pathways regulating cancer occurrence, development, and even poor prognosis, including epithelial-mesenchymal transition, hypoxia, angiogenesis, KRAS signal upregulation, interleukin 6-mediated JAK-STAT signaling pathway, and inflammatory response. Two identified pairs of transcription factors, *GRHL2* and *GATA6* and *IRF5* and *GATA3*, possibly have opposite regulatory effects on SCE_H and SCE_L, respectively.

**Conclusions:**

The identification of BLCA subtypes based on cancer stem cell gene sets revealed the complex mechanism of carcinogenesis of BLCA and provides a new direction for the diagnosis and treatment of BLCA.

## Background

Bladder cancer (BLCA), generally occurs in bladder intraepithelial cells, is the fifth most common type of cancer worldwide. Approximately 151,000 new cases of BLCA and more than 52,000 related deaths worldwide are reported annually [1–3]. Urothelial cancer is the most common type of BLCA, accounting for approximately 90% of all BLCA cases [1]. Most BLCA cases can be diagnosed at an early stage, but the rate of recurrence and progression remains high, approximately 78% of patients relapse within 5 years [4]. Using various biological detection technology, molecular typing of BLCA through genetic analysis has shown differences in drug reactivity and prognosis in patients with BLCA based on their biological heterogeneity, for example, molecular classification of the Cancer Genome Atlas Quartile [5], University of North Carolina Dichotomy [6], MD Anderson Cancer Center Trisection [7], and Lund University Quintiles [8]. Although these classification methods reveal the pathogenic mechanism of BLCA at the molecular level, they do not fundamentally demonstrate the origin of heterogeneity in tumors. New evidence suggests that cancer stem cell (CSC) subpopulations are characterized by a mixture of stem cells and cancer cells. In addition to the abilities of self-renewal and differentiation, CSCs can also act as tumors’ seeds [9, 10] and are thus considered as the driving force of heterogeneity.

Tumors are complex integrated systems composed of relatively differentiated tumor cells, infiltrating immune cells, CSCs, tumor-associated endothelial cells, stromal cells, and other cell types [11, 12]. The function and plasticity of CSCs are induced by specific signals and cell interactions in the tumor niche. Studies have shown that stem cells in melanoma can preferentially inhibit T cell activation and influence the induction of regulatory T cells, thereby evading recognition by the immune system [13]. In glioblastoma, CSCs suppress T cell responses by generating immunosuppressive cytokines through the STAT3 pathway and inducing T cell apoptosis, leading to an increase in cancer stemness and carcinogenic potential [14]. Additionally, some molecular signal transduction pathways, which control stem cell balance, are abnormally activated or inhibited to contribute to the self-renewal, proliferation, survival, and differentiation characteristics of CSCs. In a study using an experimental model of colon cancer, elevated inflammatory nuclear factor κB signal transduction enhanced Wnt activation and induced dedifferentiation of non-stem cells, which acquired tumor-initiating ability [15]. These results indicate that immune cells and their related cytokines and signal transduction pathways can directly regulate and enhance the CSC phenotype.

CD44 is a CSC surface marker [16], including BLCA stem cells [17], and its overexpression is positively correlated with BLCA tumor aggressiveness. IL-6 can regulate CD44 which is essential for the maintenance of normal stem cells. In addition, abnormal activation of the JAK-STAT signaling pathway induces tumors, while the IL6/JAK/STAT3 signaling pathway helps to maintain the plasticity of breast CSCs. Meanwhile, upon its activation, the mTORC1-STAT3 signaling pathway also helps to maintain the stemness of BLCA stem cells [18, 19]. *MYC* influences somatic cell reprogramming and controls embryonic stem cell self-renewal. Following *MYC* inactivation, tumors undergo various proliferative arrests, cell differentiation, and apoptosis, thus inhibiting tumor occurrence. In liver tumor cells, Shachaf et al. have demonstrated that *MYC* inactivation triggers stem cell differentiation, while its reactivation can restore their tumor characteristics. Therefore, although the inactivation of oncogenes restores normal cells, some cells retain their potential of becoming cancerous, possibly existing in a tumor dormant state [20]. *GATA3* influences the maintenance of BLCA stem cells. Yang et al. [21] were the first to show that the KMT1A-GATA3-STAT3 signaling pathway promotes BLCA stem cell self-renewal. KMT1A protein directly catalyzes the trimethylation modification (H3K9me3) of the 9th lysine of histone H3 in the promoter region of the GATA3 gene (− 1351 to approximately − 1172), thereby inhibiting its transcription. The GATA3 protein can directly bind to the promoter region (− 1710 to approximately − 1530) of the *STAT3* gene inhibiting its transcription. Therefore, the transcriptional repression of the *GATA3* gene, mediated by histone methyltransferase *KMT1A*, promotes the upregulation of *STAT3* expression and activation, ultimately achieving the maintenance of BLCA stem cells.

**Fig. 1:**
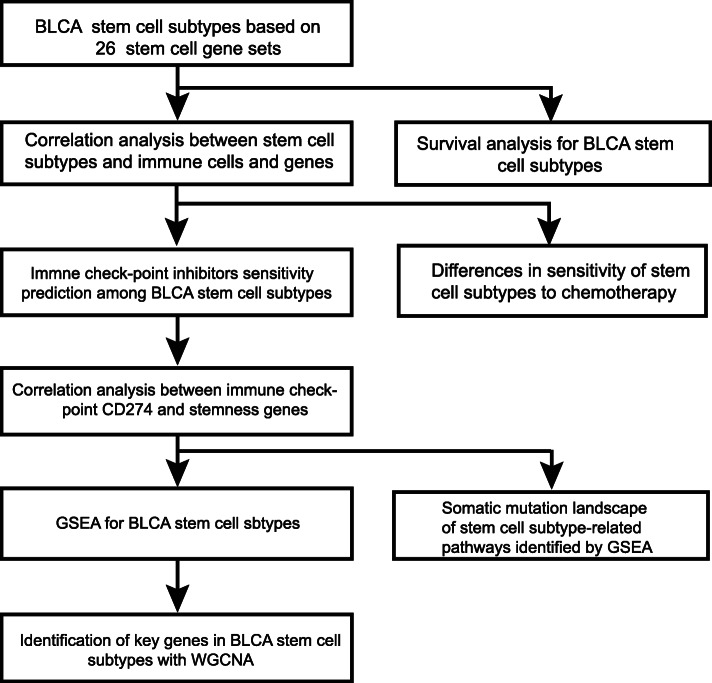
Workflow chart

In our study, we divided BLCA into high stem cell enrichment (SCE_H), medium stem cell enrichment (SCE_M), and low stem cell enrichment (SCE_L) subtypes, using stem cell gene set collection. We have demonstrated the stability and reliability of this classification with six independent datasets using the unsupervised clustering method. Importantly, we systematically examined the prognostic significance of BLCA stem cell subtypes, relationship between immune cells and genes, sensitivity of immune checkpoint inhibitor treatment, and possible changes in the biological pathways and important transcriptional regulation factors/networks (Fig. 1). Our study provides an insight into and a basis of BLCA stem cells and helps to improve clinical diagnosis and treatment of BLCA.

## Methods

### Stem cell signature collection

The 26 human stem cell gene sets used in this study were obtained from StemChecker (http://stemchecker.sysbiolab.eu/) [22]: expression checks (18), RNAi screens (1), literal curation (2), computationally derived (2), and TF target genes (3).

### Data processing

The datasets used to identify the BLCA stem cell subtypes were from three different platforms: The Cancer Genome Atlas (TCGA), Gene Expression Omnibus (GEO), and ArrayExpress databases. TCGA’s RNA-seq data (fragments per kilobase of transcript per million mapped reads (FPKM)) of 19 normal samples and 414 cancer samples, variant data of VarScan, and clinical information were downloaded from TCGA Knowledge Base (https://portal.gdc.cancer.gov/repository). Gene annotation was performed using the Ensemble database. The ArrayExpress database contains RNA-seq and clinical data (*n* = 476) for 476 cases of early urothelial carcinoma (E-MTAB-4321) FPKM from the European Genome-phenome Archive. The expression matrices of four GEO datasets, GSE13507 (*n* = 165), GSE32548 (*n* = 131), GSE31684 (*n* = 93), and GSE32894 (*n* = 308), were all quantile-normalized, and the genes were annotated in their respective platform files Illumina human -6 v2.0 expression beadchip, Illumina HumanHT-12 V3.0 expression beadchip, [HG-U133_Plus_2] Affymetrix Human Genome U133 Plus 2.0 Array, and Illumina HumanHT-12 V3.0 expression beadchip.

### Identification of BLCA subtypes based on stem cell gene sets

For each BLCA dataset, we used the GSVA package to perform a single-sample gene set enrichment analysis (ssGSEA) to quantify the enrichment level of each BLCA sample in the 26 stem cell gene sets. The ConsensusClusterPlus package was used for consensus clustering and stem cell subtype screening of the ssGSEA scores. Briefly, *k*-means clustering was performed using 50 iterations (each using 80% of samples). The best cluster number was determined by the clustering score for the cumulative distribution function (CDF) curve, and the relative changes in the area under the CDF curve were evaluated.

### Survival analysis

The Kaplan–Meier curve was used to describe the differences in survival of patients with BLCA in different datasets for classifying stem cell subtypes. We compared the survival prognosis of patients with BLCA (overall survival (OS), relapse-free survival (RFS), and progression-free survival (PFS)). The log-rank test used *P* < 0.05 as the threshold to detect significant differences in survival time.

### Immune checkpoint inhibitor treatment response prediction

Tumor immune dysfunction and exclusion is a calculation method for simulating tumor immune escape primarily by examining how the expression of each gene in the tumor interacts with the level of cytotoxic T cell (CTL) infiltration to affect patient survival [23]. We used TCGA’s FPKM RNA_seq expression profile combination subclass mapping method to predict the clinical response of BLCA stem cell subtypes to immune checkpoint blockade [24].

### Chemical response prediction

We used TCGA’s FPKM RNA seq expression profile to predict the chemotherapy response of each sample based on the largest publicly available pharmacogenomics database (Genomics of Drug Sensitivity in Cancer (GDSC), https://www.cancerrxgene.org/); six commonly used chemotherapeutic agents were selected, namely, cisplatin, doxorubicin, gemcitabine, sunitinib, methotrexate, and vinblastine. The prediction process was conducted using the R package “pRRophetic” where the half-maximum inhibitory concentration IC50 of the sample was estimated using ridge regression, and the accuracy of the prediction was evaluated using 10-fold cross-validation, according to the GDSC training set. All parameters were set to the default values, and the repeated gene expression was averaged.

### Pathway enrichment analysis

We compared the biological changes in every two subtypes in TCGA dataset and used h.all.v7.1.symbols.gmt as the reference gene set for the gene set enrichment analysis (GSEA). The analysis was performed using 1000 permutations, a < 0.05 false discovery rate (FDR) as the screening threshold, and GSEA version 4.0.1.

### Evaluation of immune cell infiltration level, tumor purity, and stromal content in BLCA

ESTIMATE was used to evaluate the level of immune cell infiltration, tumor purity, and stromal content in the BLCA stem cell typing [25].

### Comparison of immune cell fraction between BLCA stem cell subtypes

CIBERSORT is an algorithm that deconvolves the expression matrix of 22 human immune cell subgroups and can be used to estimate the proportion of immune cells [26]. We set the permutations to 1000 and used *P* < 0.05 as the screening threshold. The Kruskal–Wallis test was used to compare the differences in immune cell components of each BLCA stem cell subtype.

### Gene co-expression network analysis

To identify key genes or gene networks that characterize various stem cell subtypes in BLCA, we performed weighted correlation network analysis (WGCNA) [27] to detect gene modules associated with stem cell subtypes. The gene matrix is composed of 4876 differential genes in BLCA control normal tissues (the difference is generated by limma package in R, |log2 fold change|> 1, *P* < 0.05). WGCNA network construction and module detection used the unsigned topological overlap matrix; the best soft threshold (power) was set to 3, the minimum number of genes in the module was 50, and the branch merge interception height was 0.25. The hub gene was defined as that which has a Pearson correlation (due to the generally low value of the connection weight, the Pearson correlation was used) of greater than 0.30, with connections to at least 10 genes. The gene co-expression network was visualized using the Cytoscape 3.7.1 software. Wilcoxon tests were used to examine the expression differences of hub genes between stem cell subtypes. The results of survival analysis were divided into high and low groups based on the median expression of the transcription factor using the GEPIA (http://gepia.cancer-pku.cn/) database [28]. The log-rank test was used for survival distribution. Top 20 enrichment pathways were obtained using Metascape (http://metascape.org/gp/index.html#/main/step1).

### Statistical analysis

A comparison of the estimated IC50 of BLCA stem cell subtypes was performed using the Kruskal–Wallis test. *CD274* expression differences between stem cell subtypes were evaluated using ANOVA in R. All tests were two-tailed, and *P* < 0.05 was considered as statistically significant.

## Results

### BLCA subtypes identified based on stem cell gene sets

**Fig. 2:**
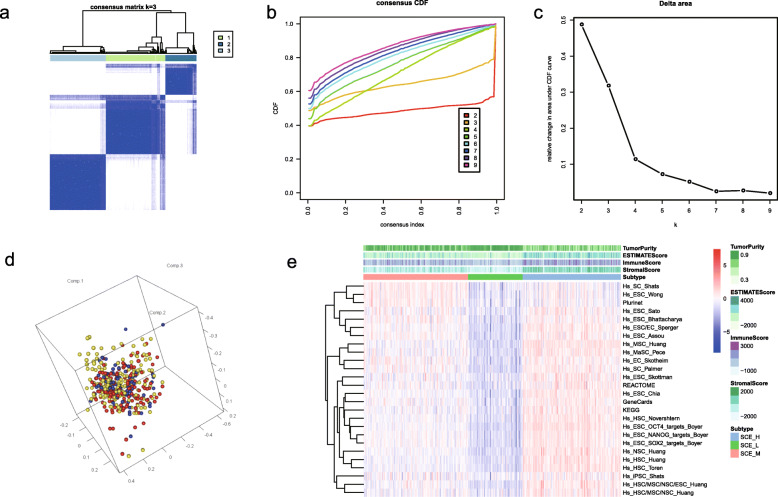
Identification of three stem cell subtypes in the BLCA TCGA cohort. **a** Consensus score matrix for BLCA samples when *k* = 3. A higher consensus score between two samples indicates that they are more likely to be assigned to the same cluster in different iterations. **b** CDF describes a real random variable of its probability distribution based on consensus scores for different subtype numbers (*k* = 2–9). **c** Delta area curve of all samples when *k* = 3. **d** Three-dimensional plot for ssGSEA scores in three stem cell subtypes; each dot indicates a sample, and different colors represent different subtypes. **e** Heatmap of ssGSEA scores for three subtypes. BLCA, bladder cancer; ssGSEA, single-sample gene set enrichment analysis; CDF, cumulative distribution function; TCGA, The Cancer Genome Atlas

We collected 26 stem cell gene sets representing unique self-regenerating properties (Supplementary Table 1) and quantified the scores of 26 stem cell gene sets in each sample using ssGSEA. We used the ConsensusClusterPlus package to divide all tumor samples into *k* (*k* = 2–9) different subtypes. The CDF curve based on the consensus scores achieves the best division when *k* = 3. Additionally, the principal component analysis results indicated that the ssGSEA scores, based on the 26 stem cell gene sets, were divided into 3 subtypes (Fig. 2a–d), which were defined as SCE_H, SCE_M, and SCE_L (Fig. 2e). Similarly, we performed the same clustering and subtyping for the remaining datasets E-MTAB-4321, GSE13507, GSE31684, GSE32548, and GSE32894 (Supplementary Figure 1).

When using ESTIMATE to evaluate the level of immune infiltration in all datasets, we found that the SCE_H immune score in all 6 datasets was much higher than that for other subtypes, and SCE_L showed the lowest immune score (Supplementary Figure 2). The comparison of the stromal content showed the same trend (SCE_H > SCE_M > SCE_L). However, the comparison of tumor purity of the BLCA stem cell subtypes showed opposite results. SCE_H and SCE_L showed the lowest and highest tumor purity (SCE_L > SCE_M > SCE_H), respectively. This is consistent with the results observed for most HLA genes and immune cell marker genes evaluated, such as *CD8A* (CD8 T cells), *GZMA* (cytotoxic cells), *IFNG* (Th1 cells), *PMCH* (Th2 cells), *CD68* (macrophages), and *IL17A* (Th17 cells), among others, which were significantly upregulated and downregulated in SCE_H and SCE_L, respectively (Supplementary Figure 3).

**Fig. 3:**
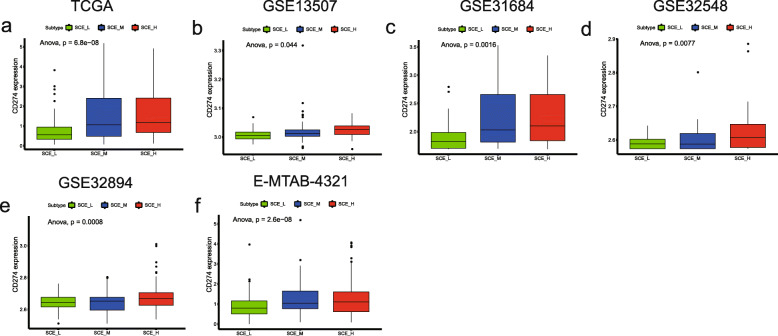
Differential expression of *CD274* in BLCA stem cell subtypes. Expression of immune checkpoint *CD274* (*PD-L1*) in **a** TCGA, **b** GSE13507, **c** GSE31684, **d** GSE32548, **e** GSE32894, and **f** E-MTAB-4321 cohort determined by ANOVA. TCGA, The Cancer Genome Atlas; ANOVA, analysis of variance

Due to the close association between BLCA stem cell subtypes and immunity, we focused on the differential expression of *CD274* (*PD-L1*) in each subtype (Fig. 3). In all six datasets, SCE_H and SCE_L showed the highest and lowest expression levels, respectively, indicating that the BLCA subtype SCE_H was more sensitive to anti-*PD-1* immunotherapy than the remaining subtypes. Subsequent immune checkpoint inhibitor treatment response prediction and survival analysis have both confirmed these results.

### Survival of patients with different BLCA stem cell subtypes

**Fig. 4:**
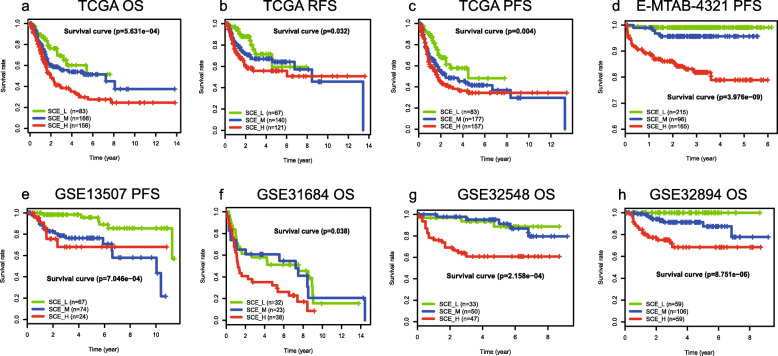
Survival analysis of patients with different BLCA stem cell subtypes. Comparison of survival prognosis between BLCA subtypes in overall survival (OS) of **a** TCGA, **f** GSE31684, **g** GSE32548, and **h** GSE32894 and relapse-free survival (RFS) in **b** TCGA together with progression-free survival (PFS) in **c** TCGA, **d** E-MTAB-4321, and **e** GSE13507. BLCA, bladder cancer; TCGA, The Cancer Genome Atlas

Since BLCA is a heterogeneous disease with a high recurrence rate, exploring the association between subtype classification and clinical prognosis is beneficial for prognosis assessment and clinical management of BLCA. We performed OS, RFS, and PFS analysis on the six datasets. Unexpectedly, all datasets showed consistent trends (Fig. 4). SCE_H and SCE_L showed the worst and best survival in the prognostic analysis (SCE_L > SCE_M > SCE_H), respectively. The *P* values of the log-rank for the OS of TCGA, GSE31684, GSE32548, and GSE32849 were 5.631e−4, 0.038, 2.158e−4, and 8.755e−6, respectively. The *P* values for the log-rank of PFS for TCGA, E-MTAB-4321, and GSE13507 were 0.004, 3.976e−9, and 7.046e−4, respectively, and the log-rank *P* value of TCGA RFS was 0.032.

### Prediction of therapeutic response of BLCA stem cell subtypes to immune checkpoint inhibitors

**Fig. 5:**
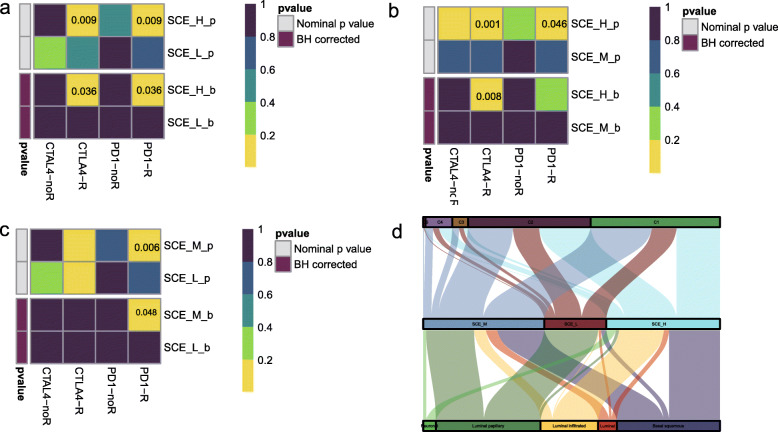
BLCA stem cell subtype immunotherapy response prediction. **a** SCE_H vs SCE_L sensitive response to *PD1* and *CTLA4* inhibitors (Benjamini and Hochberg corrected *P* = 0.036, *P* = 0.036). **b** SCE_H vs SCE_M sensitive response to *PD1* and *CTLA4* inhibitors (Benjamini and Hochberg corrected *P* > 0.05, *P* = 0.008). **c** SCE_M vs SCE_L sensitive response to *PD1* and *CTLA4* inhibitors (Benjamini and Hochberg corrected *P* = 0.048, *P* > 0.05). **d** Sankey chart showing the distribution of BLCA stem cell subtypes in C1–C6 (C5 was not available for BLCA) and molecular subtypes. BLCA, bladder cancer; SCE_H, high stem cell enrichment; SCE_M, medium stem cell enrichment; SCE_L, low stem cell enrichment

Based on the above results, we further evaluated the responses of the three subtypes to immunotherapy. At present, 5 *PD-1*/*PD-L1* immunotherapy drugs have been approved by the Food and Drug Administration for treating BLCA. This includes nivolumab and pembrolizumab (both *PD-1* inhibitors) approved in 2016 and 2017 for treating patients with locally advanced or metastatic urothelial cancer who were administered first-line platinum-containing chemotherapy for 1 year [29–34]. We used the tumor immune dysfunction and exclusion algorithm to predict the likelihood of a response to immunotherapy. The results showed significant differences in the responses to immunotherapy among the SCE_H (20%, 32/158), SCE_M (42%, 71/168), and SCE_L groups (61%, 54/88) (*P* = 2.951e−10). We further performed subclass mapping to compare the expression profiles of the three stem cell subtypes which were defined using another published dataset containing 47 patients with melanoma who responded to immunotherapy [35]. In a pairwise comparison of the three subtypes, more promising results were observed in SCE_H for the anti-*PD1* and anti-*CTLA4* treatments compared to the other subtypes (Fig. 5a–c) (anti-*PD1* therapy: SCE_H vs SCE_L, FDR = 0.036; SCE_H vs SCE_M, *P* = 0.046; SCE_M vs SCE_L, FDR = 0.048; anti-*CTLA4* therapy: SCE_H vs SCE_L, FDR = 0.036; SCE_H vs SCE_M, FDR = 0.008). We further correlated the BLCA stem cell typing results with the published molecular typing and immunotyping results in TCGA cohort. SCE_H primarily corresponded to the molecular subtypes luminal-infiltrated and basal squamous and C1 and C2 for immune subtypes; SCE_L primarily corresponded to luminal-papillary and C1–C4, and SCE_M showed a wide distribution of molecular and immune subtypes (Fig. 5d).

### Differences in sensitivity of stem cell subtypes to chemotherapy

**Fig. 6:**
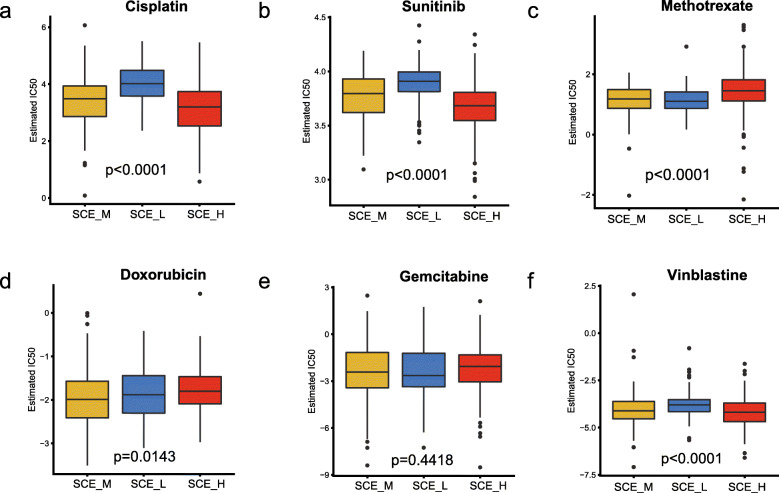
Differences in sensitivity of stem cell subtypes to chemotherapy. The box plots of the estimated IC50 for **a** cisplatin, **b** sunitinib, **c** methotrexate, **d** doxorubicin, **e** gemcitabine, and **f** vinblastine are shown for C1–C4 in TCGA cohort. *P* value in the box plots is for all groups. TCGA, The Cancer Genome Atlas; SCE_H, high stem cell enrichment; SCE_M, medium stem cell enrichment; SCE_L, low stem cell enrichment

Since chemotherapy is a common treatment strategy for patients with BLCA, we selected six chemotherapeutic agents (cisplatin, doxorubicin, gemcitabine, sunitinib, methotrexate, and vinblastine) and evaluated the response of the three subtypes. We designed the prediction model on the GDSC cell line dataset using ridge regression and evaluated the satisfactory prediction accuracy using 10-fold cross-validation. We estimated the IC50 of each sample in TCGA dataset based on the prediction models of these six chemotherapeutic agents. For cisplatin, sunitinib, and vinblastine, SCE_L was the least sensitive while SCE_H was the most sensitive compared to the other subtypes. For doxorubicin, SCE_M was the most sensitive, while for gemcitabine and methotrexate, SCE_M was the most sensitive and SCE_L was the least sensitive relative to the other subtypes (Fig. 6).

### Differences among 22 human immune cell subgroups of BLCA stem cell subtypes in CIBERSORT

To explain the difference in survival of patients with different BLCA stem cell subtypes, we used the CIBERSORT algorithm to calculate the proportions of 22 immune cells in each subtype of the six datasets, with *P* < 0.05 as the threshold for screening. The results showed that the proportions of macrophages M0, M1, and M2 had an upward trend in the SCE_H subtype (except for GSE13507) and the proportion of regulatory T cells (Tregs) was significantly (*P* < 0.05) increased in the SCE_H subtype (Supplementary Figure 4A–F). We also used the ssGSEA scores of immune cells in each cohort as continuous variables and a performed univariate Cox analysis (Supplementary Table 2). Further, we divided the median value of the corresponding data’s ssGSEA scores into groups with high and low scores. A high score indicated that patients with a high macrophage M0 content had a worse prognosis. This is consistent with the poor clinical prognosis of patients with the SCE_H subtype compared to that of patients with the other subtypes. The trend for macrophages M2 was similar to that of macrophages M0, whereas macrophages M2 and Tregs showed opposite trends (Supplementary Figure 4G–L). This indicates that compared to Tregs, macrophages M0 and M2 have completely opposite regulatory mechanisms during BLCA prognosis.

### Correlation of *CD274* with stemness genes and risk observation of stem cell subtype populations

**Fig. 7:**
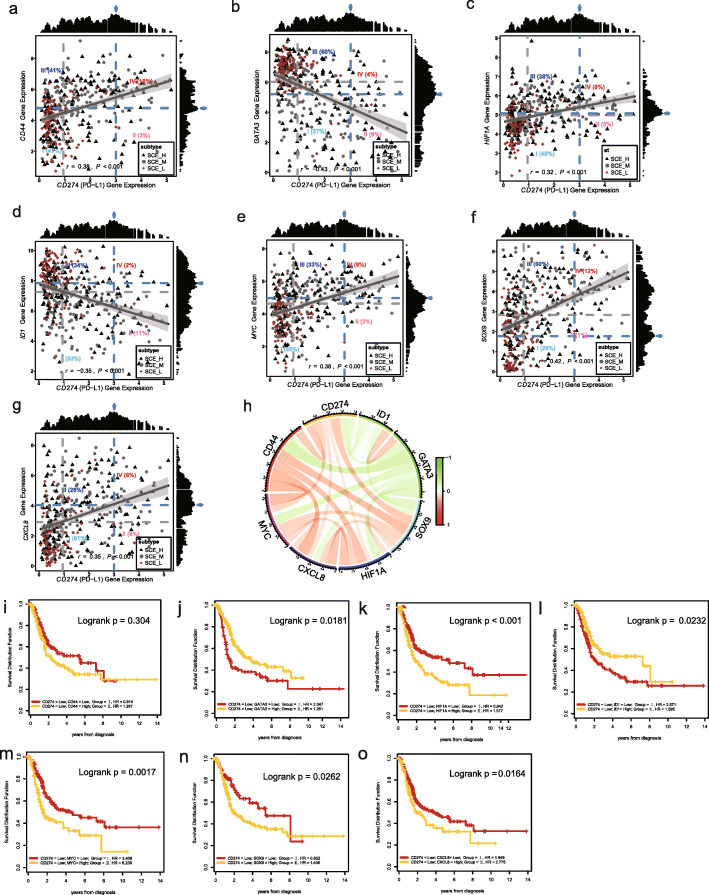
Stem cell stratification analysis of BLCA based on *CD274* and stem gene expression. The scatter plot shows the gene expression value of *CD274* and stemness genes (*CD44*, *GATA3*, *HIF1A*, *ID1*, *MYC*, *SOX9*, *CXCL8*) after log2 conversion (**a**–**g**). Log-rank test was used to determine the best cutoff value to divide continuous variables of gene expression into high and low expression groups. The blue arrow shows the highest test score of the candidate cutoff point based on the gene expression value of the log2 transformation, so the patients were divided into 4 different risk groups (blue dotted line, groups I–IV). The risk groups are those with their proportions expressed as percentages. A linear regression line was drawn; the gray-shaded area shows the 95% confidence interval, and the correlation analysis revealed the Pearson correlation coefficient. The SCE_H (black triangle), SCE_M (gray circle), and SCE_L (brown diamond) status are marked as BLCA samples. **h** Correlation diagram of *CD274* and stem genes, Pearson correlation coefficient; red indicates positive correlation, and green indicates negative correlation. **i**–**o** Kaplan–Meier survival curves of risk groups I and III were plotted, log-rank test. SCE_H, high stem cell enrichment; SCE_M, medium stem cell enrichment; SCE_L, low stem cell enrichment; BLCA, bladder cancer

We identified an important immune role for *CD274* in the stem cell subtypes, and thus, we further explored the correlation between *CD274* and the identified stemness genes. Figure 7a–g shows the scatter plots of the expression of *CD274* and stemness genes *CD44*, *GATA3*, *HIF1A*, *ID1*, *MYC*, *SOX9*, and *CXCL8* in the BLCA TCGA cohort. Among them, *CD274* was negatively correlated with *ID1* and *GATA3* and positively correlated with *CD44*, *HIF1A*, *MYC*, *SOX9*, and *CXCL8* (Fig. 7h). We divided the patients according to the optimal expression cutoff of CD274 and each stemness gene into risk groups I, II, III, and IV (for example, *CD274* and *CD44* corresponded to *CD274*^low^*CD44*^low^, *CD274*^high^*CD44*^low^, *CD274*^low^ *CD44*^high^, and *CD274*^high^ CD44^high^). According to the scatter plot, for each pair of risk groups divided by *CD274* and the optimal threshold of stemness gene expression, patients with stem cell subtypes primarily belonged to groups I and III (87–88%), while the SCE_L subtype was primarily concentrated in risk group III of the *CD274* and *GATA3* and *ID1* pairs, and in the *CD274* and *CD44*, finally, the *HIF1A*, *MYC*, *SOX*, and *CXCL8* gene pairs were concentrated in risk group I. Further survival analysis of groups I and III of each gene pair showed that patients with the higher SCE_L subtype had better survival than those with a lower SCE_L subtype (Fig. 7i–o). This is consistent with the observation that patients in the SCE_L group had the longest survival duration.

### GSEA for BLCA stem cell subtypes

To explore the biological changes caused by differences in the enrichment of stem cells, we conducted a pairwise comparison of the GSEA results for each subtype.

**Fig. 8:**
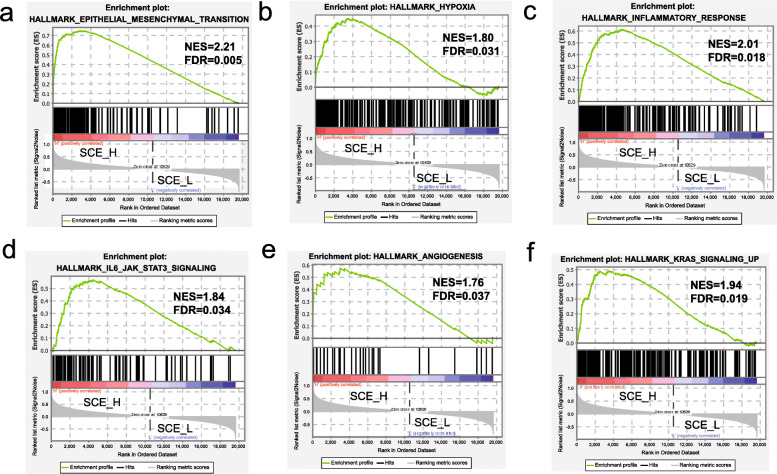
GSEA of BLCA stem cell subtypes. **a**–**f** GSEA of SCE_H vs SCE_L with GSEA hallmark as a reference gene set. GSEA, gene set enrichment analysis; SCE_H, high stem cell enrichment; SCE_L, low stem cell enrichment

By selecting at least one pathway with an FDR < 0.05, we found that as the enrichment of stem cells increased, the epithelial-mesenchymal transition (EMT) became more significant. EMT is considered as a signal of malignant transformation in all cancers, giving cells the ability to metastasize and invade, by imparting stem cell characteristics, reducing apoptosis and aging, and resisting chemical and immunotherapy [36, 37] (Supplementary Table 3). EMT can also activate multiple pathways; regulate cell metabolism, angiogenesis, proliferation, and migration; and enable cells to respond to hypoxic environments. Pathways are also significantly enriched, for example, during hypoxia, angiogenesis, inflammatory response, IL6-mediated JAK-STAT signaling pathway, and KRAS signal upregulation (Fig. 8). These pathways together constitute a vicious circle of cancer occurrence, proliferation, invasion, and metastasis.

### Somatic mutation landscape of BLCA stem cell subtypes with identified pathways

**Fig. 9:**
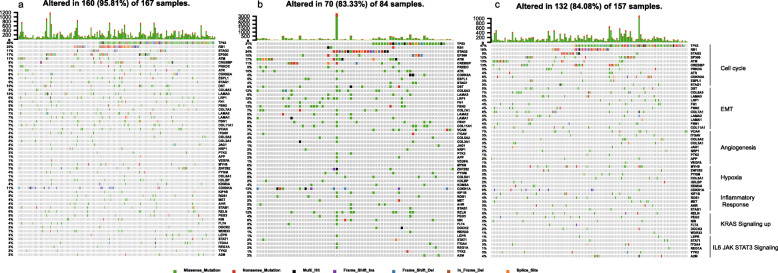
Somatic mutation landscape of stem cell subtype-related pathways identified by GSEA among **a** SCE_M, **b** SCE_L, and **c** SCE_H in TCGA cohort. SCE_H, high stem cell enrichment; SCE_M, medium stem cell enrichment; SCE_L, low stem cell enrichment

The tumor suppressor genes, *TP53* and *RB1*, play an important role in regulating cell division [38]. Inactivation, mutations, and deletions of *TP53* and *RB1* are one of the primary causes of BLCA [39]. The mutation frequency of *TP53* and *RB1* in SCE_L (31% and 4%) was much lower than that in SCE_M (56% and 25%) and SCE_H (47% and 18%). However, the mutation frequency of *STAG2*, one of the most commonly mutated genes in BLCA [40], in SCE_L (24%) was significantly higher than that of SCE_M (11%) and SCE_H (9%). In the identified EMT pathways, the mutation frequency of *COL6A3*, *LRP1*, and *FBN2* in SCE_L (12%, 12%, and 13%, respectively) was significantly higher than that of SCE_M (3%, 6%, and 5%, respectively) and SCE_H (7%, 4%, and 4%, respectively). In the hypoxia pathway, no *MYH9* mutation was observed in SCE_L (0%), while the mutation frequencies in SCE_M and SCE_H were 5% and 8%, respectively. In addition, the mutation frequencies of *CDKN1A* in SCE_L, SCE_M, and SCE_H were 13%, 11%, and 6%, respectively, and in the *KRAS* signaling pathway, the mutation frequencies of *RELN* in SCE_L, SCE_M, and SCE_H were 12%, 6%, and 5%, respectively (Fig. 9). High-frequency gene mutations during angiogenesis and inflammation and in the IL6/JAK/STAT signaling pathway were not obtained.

### Key gene networks identified in BLCA stem cell subtypes

**Fig. 10:**
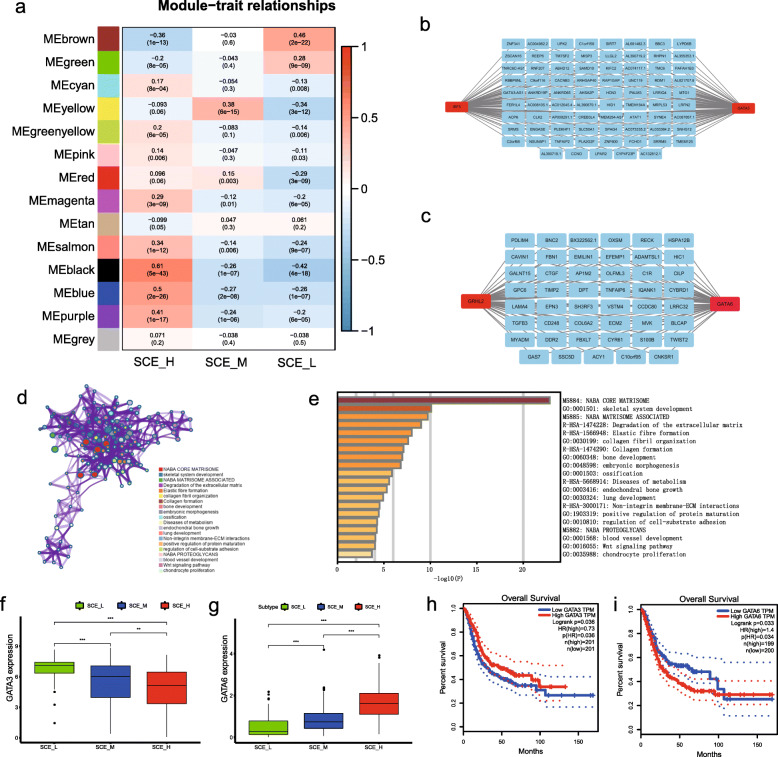
Identification of key genes/networks based on WGCNA for BLCA stem cell subtypes. **a** Correlation heat map of 14 modules in 3 stem cell subtypes obtained from 4876 differential genes compared to normal tissues and BLCA of TCGA cohort. **b** In the brown module, the co-expression network is composed of *IRF5* and *GATA3*. **c** In the black module, the co-expression network is composed of *GRHL2* and *GATA6*. **d** Top 20 enrichment pathways obtained by using Metascape in the black module. **f** Top 20 enrichment channels arranged by -Log10P value in the black module. **g** Differential expression of *GATA3* and **h** *GATA6* in BLCA stem cell subtypes, Wilcoxon tests, *P < 0.05, **P < 0.01, ***P < 0.001. **i** *GATA3* and **j** *GATA6* Kaplan–Meier survival curve according to the median expression level using GEPIA, log-rank test. BLCA, bladder cancer; TCGA, The Cancer Genome Atlas; WGCNA, weighted correlation network analysis

WGCNA is used to describe correlation patterns between genes. Using microarray gene expression data, or RNA-seq gene expression data, WGCNA can be used to identify highly correlated gene sets (module), which are randomly assigned with different colors. The colors are only used to distinguish different modules with no practical meaning or associated value. WGCNA promotes a network-based genetic screening method that can be used to identify candidate biomarkers or therapeutic targets. A total of 14 gene modules were generated based on WGCNA. Among them, brown and black modules showed the strongest association with stem cell subtypes. The brown module was positively correlated with SCE_L and negatively correlated with SCE_H and SCE_M, while the black module was positively correlated with SCE_H and negatively correlated with SCE_M and SCE_L (Fig. 10a). We intersected the genes in the black module most correlated with SCE_H and the genes in the brown module most correlated with SCE_L with human transcription factors identified using Cistrome (http://cistrome.org/). Two pairs of transcription factor regulators were identified in the brown and black modules (Fig. 10b, c), *IRF5* and *GATA3* and *GRHL2* and *GATA6*. *IRF5* is a key transcription factor regulating the differentiation of M1 macrophages into M2, enabling its anti-inflammatory role; these cells also influence tissue repair and reconstruction as well as cancer occurrence [41–43]. *GATA3* is a type 2 helper T cell (Th2) cytokine-specific transcription factor and a key stemness gene that regulates cell differentiation. It enables Th2 to express *IL-4* and other cytokines, promotes antibody production, mediates humoral immunity, and suppresses anti-tumor immunity [21, 44, 45]. Therefore, *GATA3* may act as a tumor suppressor gene in BLCA. *GRHL2* and *GATA6* play various regulatory roles during embryonic development, damage repair, epidermal barrier formation, tracheal epithelial formation, and neural tube development. They also play an important role in the occurrence and development of tumors, cell proliferation, invasion, and metastasis, which were verified by the relevant pathways identified in the black module [46–52] (Fig. 10d–e). Thus, we identified *GATA3* and *GATA6* as important transcription factors with opposite expression and effects on tumor prognosis in patients with different BLCA stem cell subtypes (Fig. 10f–i).

## Discussion

BLCA is one of the primary malignant tumors that endangers human health. Although several molecular genotyping schemes for BLCA have been proposed, they remain in their infancy stage compared with those of breast cancer [53–56]. A unified, well-developed, and highly feasible molecular typing scheme is required for better diagnosis and treatment of BLCA. Additionally, to date, no studies have classified BLCA based on stem cell gene sets, and thus, we used specific stem cell gene sets to identify and verify our new classification for BLCA. BLCA can be divided into three stable subtypes: SCE_H, SCE_M, and SCE_L. Among them, the SCE_H subtype showed the highest degree of immune infiltration and lowest tumor purity relative to the other subtypes. Patients with this subtype have the worst prognosis. This appears to contradict the previous suggestions that a higher degree of tumor immune infiltration is associated with a better prognosis. Using the CIBERSORT analysis of the immune cell fraction of stem cell subtypes, we found that the proportion of various cells associated with cytotoxicity in the SCE_H subtype was significantly lower than that in the other subtypes, such as resting NK cells, CD8 T cells, and CD4 T cells. The number of macrophages M0, M1, and M2 was significantly increased. The composition of immune cells in the tumor microenvironment is complex and has different roles in various stages of tumor progression. Among them, macrophages show the highest content in tumor tissues and had the most significant regulatory effect on tumors. These cells, which can promote the proliferation, invasion, and metastasis of tumor cells and induce tumor cells to develop immune tolerance, are known as tumor-associated macrophages (TAMs) (most studies have suggested that TAMs are primarily the M2 type) [57]. TAMs are often distributed around CSCs, and the amount of infiltration is closely correlated to the tumor histological grade and number of CSCs. Jinushi et al. [58, 59] found that the growth factor and inflammatory cytokine *MFG-E8* and *IL-6* secreted by TAM activate the *STAT3* and sonic hedgehog signaling pathways, thus inducing CSC formation and enhance CSC tumorigenesis and resistance to chemotherapy. This is consistent with our survival analysis showing high ssGSEA scores for macrophage M0 and M2 and predicting a poor prognosis for patients with BLCA.

In addition, the immune checkpoint molecule, *CD274* (*PD-L1*), was significantly upregulated in the SCE_H subtype. This molecule suppresses the proliferation and differentiation of T lymphocytes, promotes the differentiation of Tregs, and induces the secretion of cytokines, thereby suppressing the immune response [57]. Prediction of the anti-*PD-1* treatment response showed that SCE_H is more sensitive to anti-*PD-1* than other subtypes, indicating that *CD274* is highly expressed in tumor/tumor stem cells and may be involved in the tumor immune escape process. SCE_H primarily corresponds with the luminal-infiltrated and basal-squamous molecular subtypes of BLCA. These two types of tumors exhibit high levels of immune infiltration and respond well to immune checkpoint therapy (*PD-1*, *PD-L1*, and *CTLA4*). This demonstrates that the stem cell classification we defined is closely correlated with the existing molecular typing of BLCA. For locally advanced/metastatic patients, the standard first-line treatment strategy is combination chemotherapy (MVAC) consisting of methotrexate, vinblastine, doxorubicin, and cisplatin, and dual therapy (GC) consisting of gemcitabine and cisplatin [60, 61]. Compared with other subtypes, SCE_H had the highest sensitivity to cisplatin, sunitinib, and vinblastine, while SCE_L was more sensitive to methotrexate and gemcitabine than the other subtypes. Methotrexate, an anti-folate chemotherapeutic agent, inhibits tumor cell DNA synthesis by inhibiting dihydrofolate reductase, thus halting tumor growth and reproduction. This effect may be attributed to the lower mutation rate of *TP53* and *RB1* during cell cycle in the SCE_L subtypes than other subtypes. Patients of SCE_H and other subtypes of BLCA may benefit from a combination of chemotherapy and immunotherapy.

Next, we explored the biological changes caused by different levels of BLCA stem cell enrichment and showed that SCE_H was positively correlated with EMT, hypoxia, angiogenesis, and inflammatory response activation in the tumor microenvironment. Studies have confirmed that early tumor cells are in an epithelioid state, and as the tumor progresses, more mesenchymal features are gradually obtained, such mesenchymal cells are resistant to therapy. In addition, activation of EMT in tumor cells induces the initial stages of tumors, also known as the CSC state, suggesting that EMT is an integral process in the progression of all types of malignant tumors [62]. In a breast tumor progression model, Morel et al. [63, 64] showed that following activation by the Ras-mitogen-activated protein kinase pathway, EMT induction can drive breast epithelial cells to obtain stem cell and tumorigenic properties of CSC. In addition, *COL6A3* may be involved during the EMT process induced by TGF-β/Smad. *COL6A3* silencing inhibits cell proliferation and angiopoiesis [65], which is consistent with *COL6A3* having a lower mutation rate in SCE_H and SCE_M, and a higher mutation rate in SCE_L. Additionally, activation of the hypoxic pathway helps cancer cells to be more adaptable to the hypoxic environment. Under hypoxic conditions, the hypoxia-inducible factor (*HIF-1α*) pathway is activated to promote the release of vascular endothelial growth factor and platelet-derived growth factor, inducing endothelial cells from the original tumor blood vessels to proliferate, bud, and generate new tumor blood vessels, allowing tumors to invade and metastasize. Notably, immune cells infiltrating the tumor microenvironment can secrete a large number of cytokines and chemokines to promote EMT in tumor cells. Further, uncontrollable inflammatory lesions can regulate EMT in tumor cells, and a positive feedback loop can be formed between the inflammatory lesions and EMT, allowing the EMT process and uncontrollable inflammatory state to continue. These common pathways constitute a vicious circle of tumorigenesis, development, drug resistance, and poor prognosis.

## Conclusion

By identifying BLCA subtypes based on stem cell gene sets, we systematically analyzed the relationship between these subtypes in the tumor microenvironment and immune cells, immunotherapy/chemotherapy response, corresponding pathways, and key genes. These results provide a basis and reference for the clinical diagnosis and treatment of BLCA.

## Supplementary information

**Additional file 1 **: **Table S1.** The 26 stem gene sets used for identification of BLCA subtype. BLCA: Bladder cancer. **Figure S1.** Clustering heat map of stem cell subtype in (A) E-MTAB-4321, (B) GSE13507, (C) GSE31684, (D) GSE32548, and (E) GSE32894. **Figure S2.** Evaluation of immune cell infiltration level, tumor purity, and stromal content in BLCA. (A–F) Immune score, (G–L) stromal score (stromal content), and (M–R) tumor purity in all six datasets. *P < 0.05, **P < 0.01, ***P < 0.001; ns means not significant. BLCA: bladder cancer. **Figure S3.** Comparisons of the expression levels of immune-related genes between BLCA subtypes. (A–C) Expression levels of HLA genes between BLCA subtypes in TCGA, E-MTAB-4321 and GSE32894. (D–E) Expression levels of immune cell subgroup marker genes between BLCA subtypes. Kruskal–Wallis test, *P < 0.05, **P < 0.01, ***P < 0.001; ns means not significant. BLCA: bladder cancer. **Figure S4.** Difference analysis of 22 human immune cell subgroups of BLCA stem cell subtypes in CIBERSORT. Immune cell subgroups with significant differences in BLCA stem cell subtypes in (A) TCGA, (B) GSE32894, (C) GSE31684, (D) E-MTAB-4321, (E) GSE13507, and (F) GSE32548 cohort with CIBERSORT. Fraction of different immune cell subgroups among the four subtypes evaluated using Kruskal–Wallis tests, * P < 0.05, ** P < 0.01, *** *P* < 0.001. Kaplan–Meier survival curve based on median ssGSEA score for (G) TCGA, (H) GSE13507, (I) GSE32548, and (J) GSE32894, and best cut-off for (K) E-MTAB-4321 cohort in OS for macrophage M0, together with median ssGSEA score for (L) TCGA in OS for macrophage M2. BLCA: bladder cancer; TCGA: The Cancer Genome Atlas. **Table S2.** Univariate Cox analysis for all six datasets. **Table S3.** GSEA for BLCA stem cell subtypes.

## Data Availability

The datasets generated or analyzed for this study can be found in the TCGA Knowledge Base (https://portal.gdc.cancer.gov/repository), GEO (https://www.ncbi.nlm.nih.gov/geo/), and ArrayExpress (https://www.ebi.ac.uk/arrayexpress/) databases. Twenty-six stem cell gene sets were obtained from StemChecker (http://stemchecker.sysbiolab.eu/).

## Abbreviations

SCE_H: High stem cell enrichment
SCE_M: Medium stem cell enrichment
SCE_L: Low stem cell enrichment
BLCA: Bladder cancer
CSC: Cancer stem cell
TCGA: The Cancer Genome Atlas
GEO: Gene Expression Omnibus
FPKM: Fragments per kilobase of transcript per million mapped reads
ssGSEA: Single-sample gene set enrichment analysis
CDF: Cumulative distribution function
OS: Overall survival
RFS: Relapse-free survival
PFS: Progression-free survival
CTL: Cytotoxic T cell
WGCNA: Weighted correlation network analysis
ANOVA: Analysis of variance
Tregs: Regulatory T cells
OCLR: One-class logistic regression
EMT: Epithelial-mesenchymal transition
NES: Normalized enrichment score
FDR: False discovery rate
Th2: Type 2 helper T cell
TAMs: Tumor-associated macrophages
*HIF-1α*: Hypoxia-inducible factor
GSEA: Gene set enrichment analysis
