# Supplementary material for: Development and validation of a novel stem cell subtype for bladder cancer based on stem genomic profiling
Source: Stem Cell Res Ther. 2020 Oct 28;11:457. doi: 10.1186/s13287-020-01973-4 (PMC7594303; doi:10.1186/s13287-020-01973-4)
Supplement: Supplementary file 1 — Additional file 1 : Table S1. The 26 stem gene sets used for identification of BLCA subtype. BLCA: Bladder cancer. Figure S1. Clustering heat map of stem cell subtype in (A) E-MTAB-4321, (B) GSE13507, (C) GSE31684, (D) GSE32548, and (E) GSE32894. Figure S2. Evaluation of immune cell infiltration level, tumor purity, and stromal content in BLCA. (A–F) Immune score, (G–L) stromal score (stromal content), and (M–R) tumor purity in all six datasets. *P < 0.05, **P < 0.01, ***P < 0.001; ns means not significant. BLCA: bladder cancer. Figure S3. Comparisons of the expression levels of immune-related genes between BLCA subtypes. (A–C) Expression levels of HLA genes between BLCA subtypes in TCGA, E-MTAB-4321 and GSE32894. (D–E) Expression levels of immune cell subgroup marker genes between BLCA subtypes. Kruskal–Wallis test, *P < 0.05, **P < 0.01, ***P < 0.001; ns means not significant. BLCA: bladder cancer. Figure S4. Difference analysis of 22 human immune cell subgroups of BLCA stem cell subtypes in CIBERSORT. Immune cell subgroups with significant differences in BLCA stem cell subtypes in (A) TCGA, (B) GSE32894, (C) GSE31684, (D) E-MTAB-4321, (E) GSE13507, and (F) GSE32548 cohort with CIBERSORT. Fraction of different immune cell subgroups among the four subtypes evaluated using Kruskal–Wallis tests, * P < 0.05, ** P < 0.01, *** P < 0.001. Kaplan–Meier survival curve based on median ssGSEA score for (G) TCGA, (H) GSE13507, (I) GSE32548, and (J) GSE32894, and best cut-off for (K) E-MTAB-4321 cohort in OS for macrophage M0, together with median ssGSEA score for (L) TCGA in OS for macrophage M2. BLCA: bladder cancer; TCGA: The Cancer Genome Atlas. Table S2. Univariate Cox analysis for all six datasets. Table S3. GSEA for BLCA stem cell subtypes. [file 13287_2020_1973_MOESM1_ESM.zip › Supplementary_File.docx]

**Supplementary Materials**

Supplementary Table 1. **The 26 stem gene sets used for identification of BLCA subtype.** BLCA: Bladder cancer

Supplementary Figure 1. Clustering heat map of stem cell subtype in (A) E-MTAB-4321, (B) GSE13507, (C) GSE31684, (D) GSE32548, and (E) GSE32894.

Supplementary Figure 2. **Evaluation of** **immune cell infiltration level, tumor purity, and** **stromal content in BLCA.** (A–F) Immune score, (G–L) stromal score (stromal content), and (M–R) tumor purity in all six datasets. *P < 0.05, **P < 0.01, ***P < 0.001; ns means not significant. BLCA: bladder cancer

Supplementary Figure 3. **Comparisons of the expression levels of immune-related genes between BLCA subtypes**. (A–C) Expression levels of HLA genes between BLCA subtypes in TCGA, E-MTAB-4321 and GSE32894. (D–E) Expression levels of immune cell subgroup marker genes between BLCA subtypes. Kruskal–Wallis test, *P < 0.05, **P < 0.01, ***P < 0.001; ns means not significant. BLCA: bladder cancer

Supplementary Figure 4. **Difference analysis of 22 human immune cell subgroups of BLCA stem cell subtypes in CIBERSORT.** Immune cell subgroups with significant differences in BLCA stem cell subtypes in (A) TCGA, (B) GSE32894, (C) GSE31684, (D) E-MTAB-4321, (E) GSE13507, and (F) GSE32548 cohort with CIBERSORT. Fraction of different immune cell subgroups among the four subtypes evaluated using Kruskal–Wallis tests, * P < 0.05, ** P < 0.01, *** P < 0.001. Kaplan–Meier survival curve based on median ssGSEA score for (G) TCGA, (H) GSE13507, (I) GSE32548, and (J) GSE32894, and best cut-off for (K) E-MTAB-4321 cohort in OS for macrophage M0, together with median ssGSEA score for (L) TCGA in OS for macrophage M2. BLCA: bladder cancer; TCGA: The Cancer Genome Atlas

Supplementary Table 2. **Univariate Cox analysis for all six datasets.**

Supplementary Table 3**. GSEA for BLCA stem cell subtypes.**

| Pathway | NES | FDR |
| --- | --- | --- |
| HALLMARK_EPITHELIAL_MESENCHYMAL_TRANSITION |  |  |
| SCE_H vs SCE_L | 2.21 | 0.005 |
| SCE_H vs SCE_M | 2.42 | 0 |
| SCE_M vs SCE_L | 2 | 0.008 |
| HALLMARK_HYPOXIA |  |  |
| SCE_H vs SCE_L | 1.8 | 0.031 |
| SCE_H vs SCE_M | 1.41 | 0.129 |
| SCE_M vs SCE_L | 1.72 | 0.034 |
| HALLMARK_INFLAMMATORY_RESPONSE |  |  |
| SCE_H vs SCE_L | 2.01 | 0.018 |
| SCE_H vs SCE_M | 1.81 | 0.019 |
| SCE_M vs SCE_L | 1.89 | 0.015 |
| HALLMARK_IL6_JAK_STAT3_SIGNALING |  |  |
| SCE_H vs SCE_L | 1.84 | 0.034 |
| SCE_H vs SCE_M | 1.61 | 0.069 |
| SCE_M vs SCE_L | 1.71 | 0.033 |
| HALLMARK_ANGIOGENESIS |  |  |
| SCE_H vs SCE_L | 1.76 | 0.037 |
| SCE_H vs SCE_M | 1.98 | 0.004 |
| SCE_M vs SCE_L | 1.33 | 0.182 |
| HALLMARK_KRAS_SIGNALING_UP |  |  |
| SCE_H vs SCE_L | 1.94 | 0.019 |
| SCE_H vs SCE_M | 2.19 | 0.001 |
| SCE_M vs SCE_L | 1.50 | 0.087 |

NES >1.0 and FDR <0.25 were considered significant.

GSEA: gene set enrichment analysis; SCE_H: high stem cell enrichment; SCE_M: medium stem cell enrichment; SCE_L: low stem cell enrichment; NES: normalized enrichment score; FDR: false discovery rate.
